# Complete genome sequence of *Dehalococcoides mccartyi* strain NK, an acid-tolerant organohalide-respiring bacterium

**DOI:** 10.1128/mra.00633-25

**Published:** 2025-08-11

**Authors:** Yiru Cui, Yi Yang, Xiuying Li, Jingjing Wang, Huijuan Jin, Hengyi Liao, Xin Wang, Ke Shi, Siqi Huang, Tongyue Zhou, Frank E. Löffler, Jun Yan

**Affiliations:** 1 Key Laboratory of Pollution Ecology and Environmental Engineering, Institute of Applied Ecology, Chinese Academy of Sciences 74763 , Shenyang, Liaoning, China; 2 University of Chinese Academy of Sciences 74519, Beijing, China; 3 Water Resources Research Center, University of Hawaii at Manoa, Honolulu, Hawaii, USA; 4 Department of Civil, Environmental and Construction Engineering, University of Hawaii at Manoa, Honolulu, Hawaii, USA; 5 Department of Civil and Environmental Engineering, University of Tennessee, Knoxville, Tennessee, USA; Montana State University, Bozeman, Montana, USA

**Keywords:** *Dehalococcoides*, reductive dechlorination, low pH, bioremediation

## Abstract

*Dehalococcoides mccartyi* strain NK reductively dechlorinates tetrachloroethene to ethene at pH 5.5. The metagenome-assembled strain NK genome is 1.51 Mb in size with a G + C content of 48.6%.

## ANNOUNCEMENT

*Dehalococcoides mccartyi* (*Dhc*) strains are organohalide-respiring bacteria that use chlorinated organics as electron acceptors in growth-coupled reductive dechlorination reactions (1). All characterized *Dhc* strains to date are neutrophiles and grow between pH 6.0 and 8.0 (1, 2). A novel *Dhc* strain NK was enriched in a low-pH dechlorinating consortium sourced from a freshwater sediment of the Neckar River, Germany.

The consortium was cultivated at 30°C in reduced mineral salt medium (3) supplied with 0.5 mM tetrachloroethene as an electron acceptor and buffered with 60 mM 2-(N-morpholino)-ethanesulfonic acid to maintain a pH of 5.5. Transfers were made every 2–3 months following complete conversion to ethene. Biomass was harvested from 1 L culture suspension via vacuum filtration onto a 0.22 µm pore size membrane filter. Genomic DNA was extracted using the E.Z.N.A. stool DNA Kit (Omega Bio-Tek, Norcross, GA) per the manufacturer’s instructions. A metagenomic shotgun sequencing library was constructed using the Nextera XT DNA Library Preparation Kit and sequenced using the Illumina NovaSeq 6000 platform (Illumina, San Diego, CA) in paired-end 150 bp (PE150) mode. A total of 105,944,494 raw reads were quality-trimmed using Trimmomatic v0.36 (4). Contigs were generated using MegaHit v1.1.1-2-g02102e1 with the parameter “--min-contig-len 500” (5) and the *N*_50_ value was 71,607 bp. For PacBio HiFi sequencing, 5 µg of DNA was repaired using the PreCR Repair Mix Kit (New England Biolabs, Ipswich, MA) prior to library construction and then size-selected to obtain >3 kb fragments. A SMRTbell library was constructed using the SMRTbell Prep Kit (Pacific Biosciences, Menlo Park, CA), and sequencing was performed on a PacBio Sequel IIe platform (Pacific Biosciences) using a single SMRT 8M cell. The HiFi reads were generated and trimmed using SMRT Link v11.0 (Pacific Biosciences), yielding a raw read *N*_50_ value of 9,907 bp. A hybrid assembly was conducted using 2,766,415 HiFi long-reads (coverage, 16,237×) and 98,545,818 Illumina short-reads (coverage, 17,809×) with OPERA-MS v2.11-r797 (6). CheckM v1.0.3 (7) estimated a 0.62% contamination in the metagenome-assembled strain NK genome. Functional annotation was performed using the NCBI Prokaryotic Genome Annotation Pipeline v6.9 (8). Default parameters were used unless otherwise noted.

The complete genome of strain NK comprises one circular 1,514,813 bp chromosome with a 48.6 mol% G + C content. A total of 1,641 genes were annotated, including single copies of 5S, 16S, and 23S rRNA genes, 1,590 coding sequences, and 46 transfer RNAs. The 16S rRNA gene of strain NK is 100% identical to that of *Dhc* strain 195 (AF004928). The strain NK genome harbors 13 intact reductive dehalogenase subunit A (*rdhA*) genes, 5 genes involved in constitutive processes (e.g., cation/proton antiporters), and 6 chaperone genes implicated in acid tolerance. No known genes for acid stress response proteins were identified (9). These genomic features provide insights into the mechanisms enabling *Dhc* to cope with acidic environments.

## Data Availability

Strain NK genome has been deposited in GenBank under the accession number CP179927. Raw reads were deposited in the Sequence Read Archive under the accession numbers SRR31880419 (PacBio) and SRR31880420 (Illumina).
